# Paenochrobactrum pullorum Bacteremia: A Case Report

**DOI:** 10.7759/cureus.59157

**Published:** 2024-04-27

**Authors:** Pradeep Kumar Mada

**Affiliations:** 1 Infectious Diseases, Comanche County Memorial Hospital, Lawton, USA

**Keywords:** ciprofloxacin, gram-negative bacteremia, maldi-tof ms, brucellaceae, paenochrobactrum pullorum

## Abstract

*Paenochrobactrum pullorum* is a gram-negative bacterium and is not usually considered a human pathogen. This is the first case report of *P. pullorum*, highlighting the importance of understanding its clinical significance. The author reports a case of *P. pullorum* bacteremia from bilateral lower extremity wound infection.

## Introduction

*Paenochrobactrum pullorum* is a gram-negative bacterium known for its nitrogen-fixing capabilities and has been isolated from various environmental sources, including the poultry environment [1]. It is not usually considered a human pathogen, and this is the first case report of *P. pullorum* bacteremia, emphasizing the importance of understanding its clinical impact. A 46-year-old homeless male with multiple comorbidities presented with bilateral lower extremity wound infections. He was found to have *P. pullorum* bacteremia.

## Case presentation

**Figure 1:**
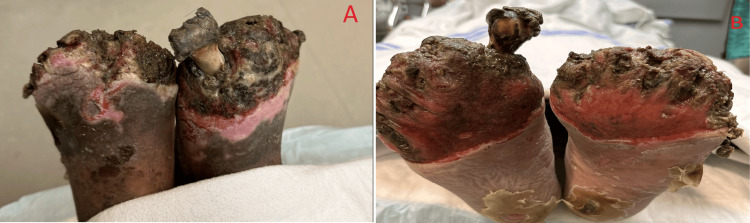
Bilateral exposed metatarsal bones due to auto amputation-necrotic stump area with a very foul-smelling purulent scanty discharge. A: dorsal view; B: plantar view

A 46-year-old homeless male with multiple comorbidities, including emphysema, bipolar disorder, schizophrenia, and a history of a motor vehicle accident in 2000 with traumatic brain injury, presented with bilateral lower extremity wound infections. He reported smoking half a pack of cigarettes per day and using marijuana but denied using alcohol or recreational drugs intravenously. He denied recent travel and exposure to animals or birds. On physical exam, his phalanges from bilateral feet came off with the socks when the staff removed his shoes (Figure 1).

He essentially had auto amputations of the trans metatarsal of both lower extremities with gangrene. There was an exposed metatarsal bone on the right lower extremity. The patient was showered, and the wounds were cleaned. In the emergency department, the patient was febrile to 102.4°F and tachycardic at 139 beats per minute. Labs revealed leukocytosis, acute kidney injury, sedimentation rate of 108 mm/hour, hemoglobin A1C of 6.1%, and lactic acid of 4.9 mmol/L (Table 1).

**Table 1:** Complete blood count and comprehensive metabolic panel results on admission.

Parameter	Results	Reference range	Units	
White blood cell count	18.81	4.40-11.00	10x3/mm^3^ (cubic millimeter)	
Red blood cell count	3.11	4.50-5.90	10x6/mm^3^
Hemoglobin (Hb)	9.1	13.2-16.5	g/dL (grams per deciliter)
Hematocrit	29	39-49	%
Mean corpuscular volume (MCV)	94	80-94	fL or fl (femtoliter)
Mean corpuscular hemoglobin (MCH)	29	27-31	pg (picograms per cell)
Mean corpuscular hemoglobin concentration (MCHC)	31	33-37	g/dL
Red cell distribution width (RDW)	14.5	11.5-16.1	%
Platelets	708	130-440	10x3/mm^3^
Mean platelet volume	8.6	7.2-11.1	fL
Sodium	137	135-145	mmol/L (millimoles per liter)
Potassium	4.5	3.5-5.0	mmol/L
Chloride	101	96-110	mmol/L
Bicarbonate	18	21-31	mmol/L
Glucose	135	80-100	mg/dL (milligrams per deciliter)
Blood urea nitrogen	33.1	6.0-21.0	mg/dL		
Creatinine	1.5	0.6-1.4	mg/dL		
Calcium	8.8	8.8-11.1	mg/dL		
Total protein	6.6	5.9-8.4	g/dL		
Albumin	2.8	3.2-5.2	g/dL		
Bilirubin total	1.35	0.00-1.20	mg/dL		
Alkaline phosphatase	145	40-129	U/L (units per liter)		
Aspartate aminotransferase	36	10-50	U/L		
Alanine aminotransferase	34	10-50	U/L		
Glomerular filtration rate (GFR)	59	60-180	mL/min/1.73 m^2^		

**Figure 2:**
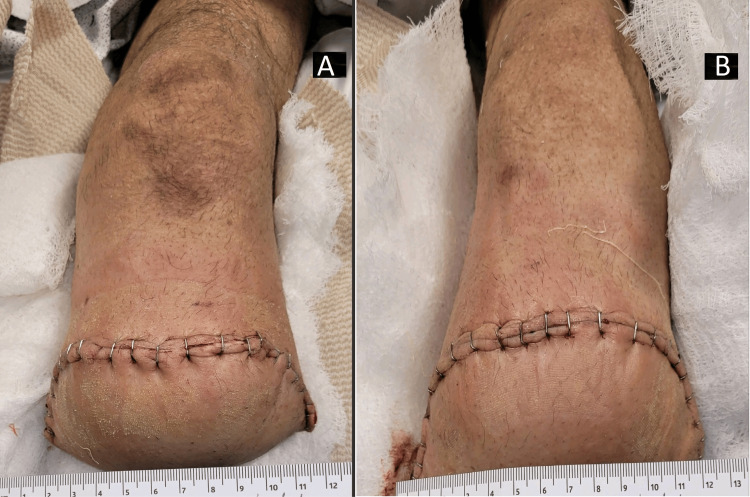
Bilateral below knee amputation (BKA). A: right BKA; B: left BKA

Blood cultures were obtained, and empiric broad-spectrum antibiotics with IV vancomycin and piperacillin/tazobactam were started. After 48 hours of incubation, two out of two blood culture bottles were positive for gram-negative rods, which were identified as *P. pullorum* (beta-lactamase negative) using matrix-assisted laser desorption/ionization time-of-flight mass spectrometry (MALDI-TOF MS) [2]. Wound cultures grew polymicrobial growth including *P. pullorum*. He underwent simultaneous bilateral below-knee amputations (Figure 2).

Histopathology of the below-the-knee amputation revealed acute osteomyelitis, active ulcers, large vessels with calcification within the wall, and viable skin, soft tissue, and bone at the margin. The sensitivity report of *P. pullorum* was not available and the patient was treated with oral ciprofloxacin 500 mg twice daily for two weeks. He was discharged in a stable condition (Table 2).

**Table 2:** Complete blood count and comprehensive metabolic panel results upon discharge.

Parameter	Results	Reference range	Units
White blood cell count	7.36	4.40-11.00	10x3/mm^3^
Red blood cell count	3.61	4.50-5.90	10x6/mm^3^
Hemoglobin (Hgb)	10.5	13.2-16.5	g/dL
Hematocrit (Hct)	34	39-49	%
Mean corpuscular volume (MCV)	95	80-94	fL
Mean corpuscular hemoglobin (MCH)	29	27-31	pg
Mean corpuscular hemoglobin concentration (MCHC)	31	33-37	g/dL
Red cell distribution width (RDW)	15.6	11.5-16.1	%
Platelets	603	130-440	10x3/mm^3^
Mean platelet volume (MPV)	8.6	7.2-11.1	fL
Neut%	62	40-74	%
Lymph%	22	19-48	%
Mono%	7.5	3.4-10.0	%
Eos%	3.8	0.0-7.0	%
Baso%	1.4	0.0-1.5	%
Neut#	4.6	1.9-8.0	10x3
Lymph#	1.6	1.0-4.9	10x3
Mono#	0.6	0.2-1.0	10x3
Eos#	0.3	0.0-0.7	10x3
Baso#	0.1	0.0-0.2	10x3
Sodium	135	135-145	mmol/L
Potassium	4.9	3.5-5.0	mmol/L
Chloride	103	96-110	mmol/L
Bicarbonate	24	21-31	mmol/L
Glucose	94	80-100	mg/dL
Blood urea nitrogen	25.8	6.0-21.0	mg/dL
Creatinine	0.7	0.6-1.4	mg/dL
Calcium	8.5	8.8-11.1	mg/dL
Total protein	6.2	5.9-8.4	g/dL
Albumin	2.7	3.2-5.2	g/dL
Bilirubin total	0.35	0.00-1.20	mg/dL
Alkaline phosphatase	77	40-129	U/L
Aspartate aminotransferase	27	Oct-50	U/L
Alanine aminotransferase	36	Oct-50	U/L

## Discussion

*P. pullorum* is a gram-negative bacterium belonging to the genus *Paenochrobactrum*, which falls under the family *Brucellaceae*. It was first described in 2010 by Kämpfer et al., after isolating from the poultry environment [3]. The genus *Paenochrobactrum* consists of three classified species including *Paenochrobactrum gallinarii*, *Paenochrobactrum glaciei*, *P.* *pullorum*, and unclassified *Paenochrobactrum* including *Paenochrobactrum *sp., *Paenochrobactrum *sp. BAB-4388, *Paenochrobactrum *sp. BAB-4391, *Paenochrobactrum *sp. DB-12, *Paenochrobactrum *sp. VRE47B1_13_1E, *Paenochrobactrum *sp. VRE69B1_13_1E [4]. ​*P. pullorum* is a rod-shaped, non-spore-forming, non-motile, oxidase-positive, and has a specific ability to fix atmospheric nitrogen, thus having a potential role in agriculture [5-7]. *P. pullorum* infections are uncommon but can arise, particularly in patients with comorbidities or exposure to environmental sources sheltering the bacterium. Though it is typically susceptible to ciprofloxacin and trimethoprim-sulfamethoxazole, appropriate antibiotic selection guided by susceptibility testing is essential for successful outcomes [3]. There is limited information available on the pathogenic potential of *P. pullorum* in humans and more research is needed to understand its pathogenic mechanisms and clinical significance in human disease.

## Conclusions

This case report highlights the significance of considering unusual pathogens like *P. pullorum* in the differential diagnosis of bacteremia, particularly in patients with environmental exposures. Timely identification and appropriate antibiotic therapy are essential for successful outcomes.
